# Simultaneous Presentation of a Right Littre's Hernia and a Left Amyand's Hernia in a School-Aged Patient

**DOI:** 10.1155/2019/4217329

**Published:** 2019-06-10

**Authors:** Andrés Eduardo Velásquez-Bueso, Luis Enrique Sánchez-Sierra, Sergio David Villeda-Rodríguez, Roberto Antonio Martínez-Quiroz

**Affiliations:** ^1^National Autonomous University of Honduras, Tegucigalpa, Honduras; ^2^Honduran Institute of Social Security, Tegucigalpa, Honduras; ^3^Pediatric Surgery Service, Hospital Escuela, Tegucigalpa, Honduras

## Abstract

**Introduction:**

Inguinal hernia is the most common condition in both male and female subjects. Amyand's hernia is characterized by the presence of the cecal appendix, swollen or not, inside the inguinal hernia sac. It is a rare condition and represents 1% of all the inguinal hernia pathology. Littre's hernia (LH) is a condition defined by the presence of a Meckel diverticulum (MD) inside a hernial sac. It is an extremely rare condition, with less than 50 cases reported in the last three hundred years, present in only 1% of all diagnosed MD.

**Case Presentation:**

A six-year-old male patient presented with a history of two bilateral protruding masses in the inguinal-scrotal region that have continued to grow since birth. No gastrointestinal symptoms were reported. Physical examination showed a bilateral inguinoscrotal mass which increased in size during the Valsalva maneuver. Surgical intervention was carried out with a bilateral hernia repair being performed under an anterior method, the surgical invagination of the MD within the small intestine and the appendix within the caecum.

**Conclusion:**

Both entities should be considered as a differential diagnosis when it comes to a pediatric patient with unilateral or bilateral inguinal hernias with an uncertain etiology, allowing an early diagnosis and prompt treatment. We present here the first recorded case of both Amyand's hernia and Littre's hernia presenting simultaneously in a pediatric patient.

## 1. Introduction

The word hernia comes from the Greek words “*epivos*” (excrescence) and “*hira*” (intestine) [1]. A hernia is the partial or complete protrusion of the gut through an abdominal wall defect, and inguinal hernias are the most common presentations across all genders. Amyand's hernia (AH) is a subclassification characterized by the presence of the cecal appendix, either swollen or not, inside the inguinal hernia sac [2, 3]. This presentation is more frequent in male patients, both adults and children [2]. It represents 1% of all the inguinal hernia pathology, and its finding is a rare condition for which reported cases are unusual [2–4]. The first recorded description of a case of this kind was by René Jacques Croissant de Garengeot in 1731. He described the presence of the cecal appendix inside the sac of a femoral hernia and named it after himself as Garengeot's hernia. In 1735, Claudius Amyand reported the first case of AH in an 11-year-old male patient with an enflamed cecal appendix inside an inguinal hernia sac [5].

Littre's hernia (LH) is a similar condition but is defined by the presence of a Meckel diverticulum (MD) inside a hernial sac as opposed to the cecal appendix [1, 6, 7]. It is an extremely rare condition, with less than 50 cases reported in the last three hundred years [8], and present in only 1% of all cases of MD, which is itself a remnant of the omphalomesenteric duct, containing all the layers of the intestinal wall with the histological peculiarity of ectopic gastric and pancreatic mucosa [9].

The MD was defined by Fabricius Hildanus in 1598, and in 1700, the French surgeon Alexis de Littré published the first case of an MD, referring to it as “an ileal diverticulum consequence of the traction made on the ileum.” It was not until 1809 that Johan Friedriech Meckel described the existing relationship between the MD and the omphalomesenteric duct [1]. The term LH was established by Reinke in 1841, as he reported the presence of a “Meckel's diverticulum inside any hernial sac” [9].

Both conditions mentioned in Introduction, AH and LH, occur very rarely and are reported in even fewer instances. We present the incidental finding and prompt treatment of a patient presenting with a right Littre's hernia and a left Amyand's hernia simultaneously found during routine bilateral inguinoscrotal hernia repair. The combination of two separate conditions makes this a unique study that should be recorded in detail.

## 2. Case Presentation

A 6-year-old male patient weighing 16.6 kg and with a height of 41 inches, from a rural location in Honduras, was evaluated by a pediatric surgeon. The subject presented with a history of two bilateral protruding masses in the inguinal-scrotal region that have grown bigger since birth. The mother denied past history of asthma or gastrointestinal symptoms such as diarrhea, melena, bloody stools, cramps, or pain, and the patient's bowel movements were normal. According to what was stated by the subject's mother, there was no family history of inguinal hernias. The patient comes from a setting with extreme poverty conditions. He had no history of previous surgical interventions.

**Figure 1:**
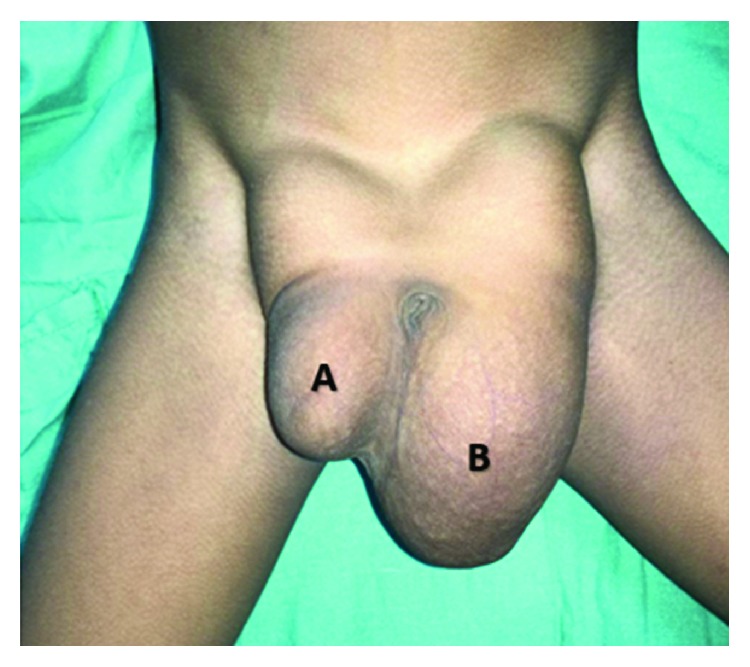
Inguinoscrotal bilateral reducible hernia: (a) right and (b) left.

During a physical examination, a bilateral inguinoscrotal mass was observed that grew bigger during the Valsalva maneuver. To palpation, masses were reducible with no tenderness. The right mass dimension was 3.1 inches × 1.5 inches and the left mass was 5.5 inches × 1.9 inches (as shown in Figure 1); testicular transillumination was negative. Bowel sounds were noticed through auscultation; however, as lab values were within normal parameters and setting conditions made it impossible to perform image studies, none was made.

Diagnostic challenges were encountered, including extreme poverty conditions and poor healthcare coverage, which made early diagnosis difficult.

Through physical examination, the patient was admitted for bilateral inguinal hernia routine surgery, most likely with visceral uncomplicated content of both hernias. Amyand's and Littre's hernias were diagnosed incidentally during surgery, after the content of both hernias were exposed.

**Figure 2:**
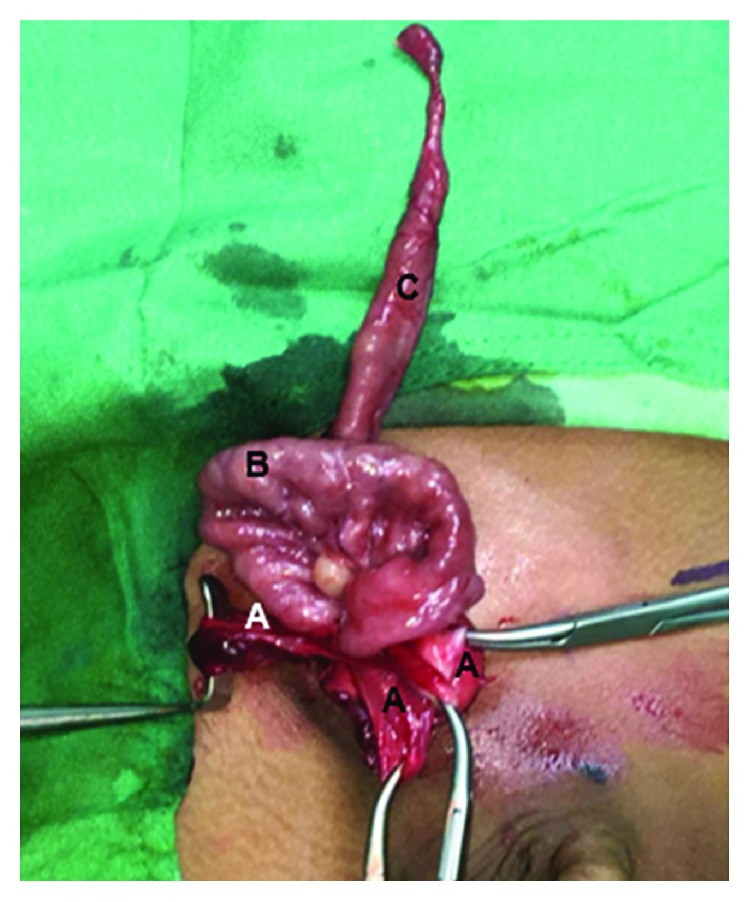
Right Littre's hernia: (a) hernial sac; (b) distal ileum; (c) Meckel's diverticula.

**Figure 3:**
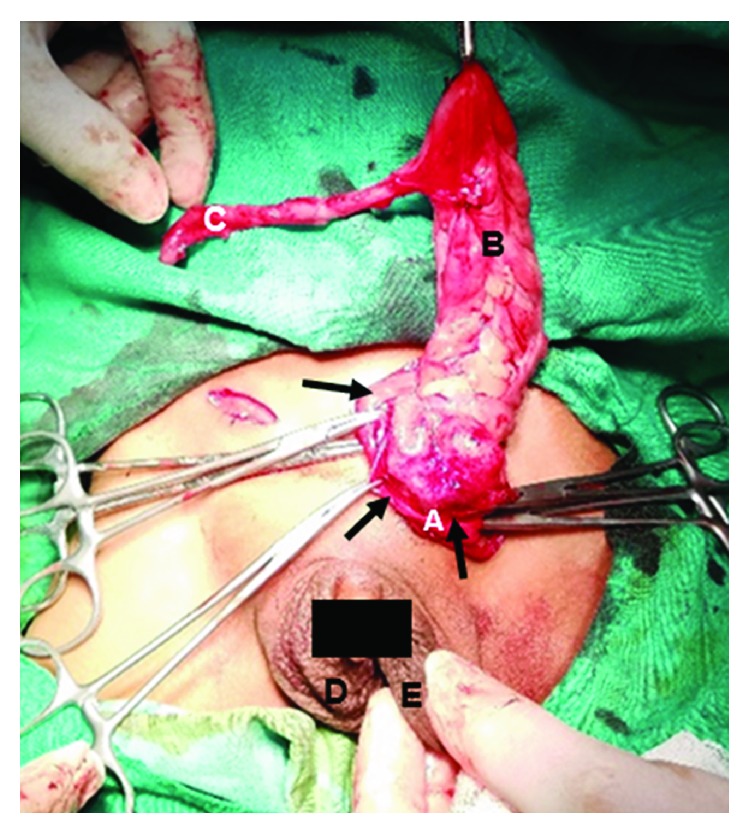
Surgical procedure: (a) Meckel's diverticula invagination; (b) cecal's appendix invagination.

**Figure 4:**
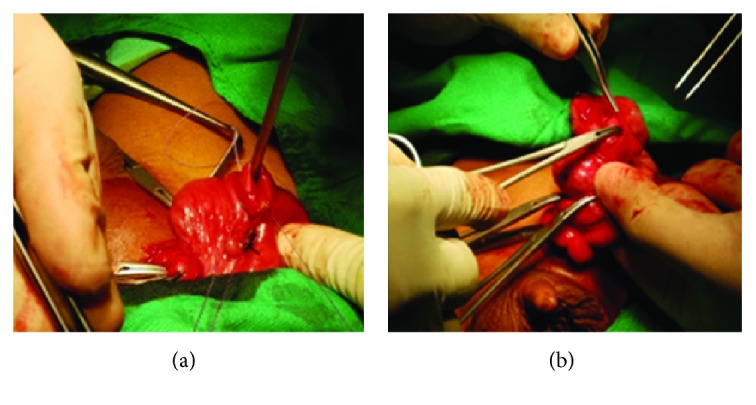
Left Amyand's hernia: (a) hernial sac; (b) caecum; (c) vermiform appendix; (d) right scrotum; (e) left scrotum.

Treatment was surgical, with bilateral hernia repair using an anterior approach. A transverse incision was made at each inguinal canal; Camper's, Scarpa's, and the external oblique fascia were dissected until the internal inguinal ring was exposed. The anterior hernial sac was grasped and secured, while the spermatic chord and the vas deferens were separated from the hernial sac. Afterwards, the hernial sac was pinched and dissected. Exploration of the right hernial sac was performed and was found to contain an MD. Its diameter was 1.3 cm (Figure 2), which was posteriorly surgically inverted into the small intestine (Figure 3). The left inguinal canal was also explored, with the cecal appendix being found inside the hernial sac. The cecal appendix was not inflamed, as can be seen in Figure 4; therefore, surgical inversion of the appendix into the colon was completed (Figure 3). No complications were reported during this procedure and the patient was stable through recovery.

## 3. Discussion

Although both hernia presentations were dealt with in the same instance, for clarity, they will initially be discussed in isolation.

### 3.1. Amyand's Hernia

As stated previously, AH is characterized by the presence of the cecal appendix, inflamed or not, inside any hernial sac. It is more frequent in men and almost exclusive to the right side [4, 10] due to the anatomical location of the cecal appendix [11]. There are exceptions to this in which AH has been reported on the left side, but this is a rare condition possibly associated with one of four options: situs inversus, intestinal malrotation, mobile caecum, or a longer cecal appendix [2]. In our patient, a mobile caecum may have caused the presence of the cecal appendix inside the hernial sac, as the other possible causes mentioned were ruled out during surgery.

Most reported cases present with an ongoing appendicitis with the hernial sac, along with features of bowel obstruction or incarceration [11]. Preoperative diagnosis of AH is difficult and image studies are only performed in rare cases, as it is a clinical condition. Despite this, preoperative computerized tomography (CT) of the patient's abdomen has been shown to help elucidate the correct diagnosis [11]. Unfortunately, the resources required for CT were not available at the hospital in question; therefore, the diagnosis was made in accordance with clinical findings.

The therapeutic approach for AH is dependent on presentation. An inflamed appendix requires immediate surgical excision. Where surgical excision is not possible, a different approach should be taken, namely, surgical inverting of the organ [11]. While this procedure has been described repeatedly in adult patients, at the time of writing, there is no standardized approach. Some efforts to fill this knowledge gap have been carried out by Losanoff and Basson in their categorization of the AH approach [12]. In the case being discussed, a surgical inverting of the cecal appendix was performed resulting in a clean, uncontaminated surgery greatly reducing the risk of peritoneal infection, thus increasing the chances of a positive clinical outcome for the patient.

### 3.2. Littre's Hernia

The Meckel diverticulum is the most common congenital gastrointestinal abnormality. It is a consequence of the persistence of the omphalomesenteric duct, which is usually obliterated after the fifth week of gestation [13, 14], with an incidence of 0.3-3% [8, 15, 16]. MD is 3-4 times more prevalent in males [17] and silent in up to 90% of cases [15]. In children, MD is typically located in the antimesenteric border of the distal ileum, 30-60 cm distal to Bauhin's valve [17]. Intestinal bleeding presents in about 55% of pediatric patients and the most common cause is related to the heterotopic gastric mucosa of the MD, implicated in 90% of cases [1, 15, 16]. Our patient presented with an MD in the same anatomic location but without clinical features or complications, and it was an incidental finding during the right scrotal inguinal hernia repair.

Littre's hernia is an extremely rare complication of an MD, present in only 1% of all cases [9, 15]. While the anatomical location of an LH can vary, most frequently, the right inguinal region is affected (50% of cases), followed by the femoral region (20-30% cases) and umbilical region (20-30% of cases). Sporadic cases in other locations such as ventral and obturator hernias have also been documented and make up 10% of cases [8, 14, 16–18]. In children, most of the cases are usually umbilical [19], but our patient presented with a right scrotal inguinal LH, which correlates with the literature. Most cases of LH become symptomatic before two years of age, while in older children clinical findings might relate with those of an acute abdominal hernia [1]. Our subject presented with none of the gastrointestinal symptoms which are expected with an LH clinical presentation. A possible explanation for this is that incarcerated LH symptoms are usually less severe and late in onset because in the early stages of the disease only the diverticulum is compromised and not the small intestine itself [8].

Clinical presentation of LH is the same as other hernial conditions, making its identification complex without the support of appropriate image studies, such as an ultrasound, which would assist in avoiding unnecessary surgical interventions. A study published by Augestad et al. suggested the use of scintigraphy, US, CT, and Technetium 99 in combination as a diagnostic tool. Unfortunately, the study was not of sufficient sample size to establish a diagnostic standard [14]. Another study conducted by Luengas et al. concluded that preoperative imaging studies do not allow the identification of an MD in the hernial sac; therefore, a diagnosis of LH will be made during surgery [1]. In contrast, a study by Messina et al. suggested that preoperative diagnosis through imaging studies might be impossible. As a result, LH diagnosis should be considered every time the physician faces an unreducible hernia [9] or indeed when faced with a reducible hernia.

In the region where the case study took place, socioeconomic conditions limit the availability of imaging equipment such as ultrasonography and CT scanning. Because of this and the previously described controversy surrounding the utilization of imaging studies, the authors prefer to base their treatment decisions on our clinical findings allowing opportune treatment for the patient. Our approach was initially to complete a right inguinal hernia repair, but incidentally, we were presented with an uncomplicated LH.

### 3.3. Surgical Approach

Whenever LH and MD are symptomatic, a surgical intervention is required. Diverticulectomy and intestinal resection with primary anastomosis are recommended when there is inflammation at the base of the diverticula, presence of palpable ectopic tissue, ischemia, or perforation [14, 17]. Our subject remained asymptomatic and did not have any complications, and therefore, resection or anastomosis was not deemed necessary.

Even though it has been reported that surgical inversion of an MD within the small intestine has been related with intussusception, it accounts for only 4% of all cases presenting with intestinal obstruction due to intussusception [20]. Due to the socioeconomic conditions of the subject, no hospitalization would have been possible in the case of a postsurgery infection; therefore, surgical inversion of the MD provided the subject with a better outcome regarding abdominal infection, a possible consequence of the resection with anastomosis.

In children, the surgical treatment for inguinal hernia (IH) is limited to division and ligation of the hernial sac at the internal inguinal ring without narrowing the ring [21]. The standard open technique with an anterior approach, used in unilateral hernia repair, as described by Le Roux et al. [22], has been reported to have no significant differences regarding hospital stay, time to resume full activity, recurrence rate, and complications such as hydrocele, wound infection, scrotal edema, erythema, and testicular atrophy. However, this approach was found to be inferior compared to a laparoscopic approach regarding bilateral pediatric inguinal hernia (PIH) repair [23].

Current trends favour laparoscopic and minimally invasive approaches, as they offer numerous advantages over open surgery for pediatric inguinal hernia repair, such as the opportunity to visually inspect the contralateral canal for the presence of an occult hernia without incision, superior visualization to potentially avoid trauma to the vas deferens and spermatic vessels, and opportunity to accomplish a safe high ligation of the sac at the internal ring [24]. Despite these advantages, the socioeconomic limitations at play meant that such an approach was not feasible. No doubt, laparoscopic PIH is a novel, safe, and elegant procedure with many advantages. Nevertheless, establishing and running a facility with this equipment may be unviable in rural settings, where the majority of the developing world resides [25].

## 4. Conclusion

AH and LH should be considered as a differential diagnosis when it comes to a pediatric patient with unilateral or bilateral inguinal hernias with an uncertain etiology, allowing an early diagnosis and prompt treatment. AH and LH are infrequent conditions typically occurring in isolation. To date, there have been no cases reported in the medical literature of both AH and LH presenting concurrently. We therefore conclude to the best of our knowledge that this is the first pediatric case reported with the association of both conditions. It is also important to acknowledge that therapeutic approaches within areas with limited resources may not allow adherence to the standardized surgical practices of developed countries.
